# The Effect of the Climatic Housing Environment on the Growth of Dairy-Bred Calves in the First Month of Life on a Scottish Farm

**DOI:** 10.3390/ani11092516

**Published:** 2021-08-27

**Authors:** David J. Bell, Jamie Robertson, Alastair I. Macrae, Amy Jennings, Colin S. Mason, Marie J. Haskell

**Affiliations:** 1 Department of Animal and Veterinary Sciences, Scotland’s Rural College (SRUC), Edinburgh EH9 3JG, UK; Colin.Mason@sruc.ac.uk (C.S.M.); Marie.Haskell@sruc.ac.uk (M.J.H.); 2 Roslin Institute, Royal (Dick) School of Veterinary Studies, University of Edinburgh, Edinburgh EH25 9JG, UK; A.I.Macrae@ed.ac.uk (A.I.M.); amy.jennings@ed.ac.uk (A.J.); 3 Livestock Management Systems Ltd., Pioneer House, Aberdeen AB11 5DE, UK; J.Robertson@lms2004.co.uk

**Keywords:** pre-weaned calf, housing, housing environment, lower critical temperature, daily liveweight gain

## Abstract

**Simple Summary:**

The climatic environment within calf housing can have an effect on calf health, but also on growth and performance. Calves have a lower threshold environmental temperature (lower critical temperature, LCT), below which can impact on the calf’s ability to maintain its core body temperature. This can cause the calf to partition more of its available energy into heat production and less into growth. The LCT decreases as the calf gets older. This year-long study followed 299 dairy-bred calves on one farm in Scotland from birth until approximately 28 days of age, and looked at the proportion of time for which the temperature was below the LCT for the individual calf, as well as the daily liveweight gain (DLWG; kg/d) of the calves during this time. For their first 6–14 days of life the calves were individually housed, and then subsequently group housed. Air temperature (°C), relative humidity (%), and wind speed (m/s) were recorded every hour of every day throughout the study, and calves were weighed regularly so that DLWG could be calculated. The study demonstrated that calves that spent a high proportion of their time below their LCT had a lower DLWG compared to calves that spent a low proportion of their time below their LCT.

**Abstract:**

Calf housing is naturally thermodynamic, with interactions between various elements such as wind speed, air temperature, and humidity. This study investigated the effect of the proportion of time for which calves were exposed to effective environmental temperatures below their lower critical temperature (LCT) on their daily liveweight gain (DLWG) within their first month of life. This study used the naturally occurring climatic environment, whereas other such studies have been conducted under climatically controlled conditions. Air temperature (°C), relative humidity (%), and wind speed (m/s) were recorded within the calf housing from birth until approximately 28 days of age, with calves being health-scored and weighed at regular intervals. Calves were housed from birth until 6–14 days old in individual hutches, and then moved into group housing igloo pens. Whilst individually housed, calves that spent less than 32% of their time below their LCT had a DLWG of 0.06 ± 0.34 kg/d (mean ± SE) compared to calves that spent more than 97% of their time below their LCT, which had a DLWG of −0.19 ± 0.045 kg/d. When group housed, calves that spent less than 1% of their time below their LCT had a DLWG of 0.59 ± 0.18 kg/d, whereas calves that spent more than 28% of their time below their LCT had a DLWG of 0.53 ± 0.23 kg/d. The proportion of time for which calves were exposed to effective environmental temperatures below their LCT had a significant effect on DLWG when calves were individually housed. Therefore, exposure to effective environmental temperatures below the LCT can be detrimental to the growth of the calf in the early stages of its life.

## 1. Introduction

For the farmer, the main objective of rearing calves is to produce a healthy calf that is able to achieve target growth rates as economically as possible. It has been well documented that poor performance in an individual calf in the pre-weaning phase can affect its future productivity [1,2,3,4,5]. Therefore, calf growth—specifically, daily liveweight gain (DLWG)—is a key performance indicator for monitoring success in calf rearing. DLWG is influenced by colostrum provision [6,7], ongoing nutrition, incidence of disease, and the quality of the calf’s environment [8,9].

Following the birthing process, calves in the UK are exposed to an environmental temperature that is typically below their thermal comfort zone (TCZ), with the most recent summary data available from the Met Office (from 1981 to 2010) showing that there is a mean annual maximum temperature of 12.4 °C and a mean annual minimum temperature of 5.3 °C [10]. The TCZ is defined as the environmental temperature at which the calf is not motivated to perform any thermoregulatory behaviour, and lies within the thermal neutral zone (TNZ) for the calf. The TNZ in the first week of life is estimated to be in the range of 15–25 °C [11]. An environmental temperature below the lower boundary of the TNZ is regarded as the lower critical temperature (LCT). Below the LCT is the point when the calf increases metabolic heat production to maintain thermal balance, which can be done by contraction of the skeletal muscles (shivering) or through non-thermogenic processes, such as increasing energy intake. The calculation of the LCT can be expressed as follows (Equation (1)):

T_lc_ = (T_r_ + H_e’min_·I_e_) − H_min_ (I_t_ + I_e_) (1)

where T_lc_ represents the lower critical temperature, T_r_ is the rectal temperature (°C), I_e_ represents external insulation (the resistance to heat loss via the skin provided by the hair coat) (°C·m^2^/watts), H_e’min_ is the minimum evaporative heat loss (watts/m^2)^, H_min_ is the thermoneutral heat production (watts/m^2^), and I_t_ represents the insulation provided by tissue (°C·m^2^/watts) [12]. Studies have investigated the effect of temperature on growth in calves. Cockram and Rowan [13] carried out a study using calves less than four weeks of age in controlled environmental chambers of 10 °C and 25 °C. They found that calves housed within the chambers at 10 °C had lower liveweight gains at 17–22 days of age than the calves housed in the chambers at 25 °C. Broucek et al. [14] reported that there was only a slight decrease in DLWG in calves that were exposed to high temperatures (average of 26.5 °C) compared to those exposed to temperatures regarded by the authors as moderate (averages of 19.5 °C and 15.7 °C). These high temperatures were recorded between June and September, which are regarded as summer and early autumn months in the Northern Hemisphere. Additionally, various studies have indicated that the season of birth can influence DLWG in calves. Place et al. [8] found from their study conducted in Pennsylvania, US, that there was a tendency for calves born in winter to have higher average DLWG than calves born in the other seasons of the year. This result was also found from the study carried out in Minnesota, US, by Chester-Jones et al. [5], where calves born in the autumn and winter gained 0.66 kg/d compared to 0.62 kg/d in calves born in summer. Therefore, season of birth and temperature are confounded, as lower temperatures would be expected during the winter months and higher temperatures during the summer months. As well as temperature and season of birth, Kelly et al. [15] highlighted the effect of relative humidity (RH) levels on DLWG; they examined the DLWG of calves housed at two temperatures (7 °C and 15 °C) and two levels of RH (75% and 95%), and found that there was no difference in the DLWG between the RH levels when housed at 15 °C; however, there was a significant increase in DLWG when housed at 7 °C with 75% RH compared to 7 °C with 95% relative humidity levels. This demonstrates that there is more than one aspect of the thermal environment that can influence DLWG.

The consumption of milk provides energy and protein to support growth, but the energy can be diverted to other metabolic processes if required. A study by Rosenberger et al. [16] showed that giving calves a higher milk volume allowance resulted in a higher weight gain; however, a compensatory mechanism for calves that are exposed to temperatures below the LCT is to increase feed consumption if additional feed is available [17]. There is also evidence to show that nutrition and the environmental temperature can affect daily liveweight gain [13] and, therefore, it is generally advised that calves are provided with more energy when the environmental temperature falls below the LCT [18,19] in order to allow the calf to partition some of this energy towards growth as well as the heat production required for maintenance. This increase in energy can be achieved by methods such as increasing the volume of milk offered or the concentration calf milk replacer (CMR) fed to the calf. The practice of feeding dairy calves milk replacer is common in the UK [20], as it is a consistent product in terms of fat, protein, and energy content, in contrast to whole milk. As would be expected, various studies have shown that increasing the quantity of CMR fed each day increases calf DLWG [21,22,23].

The objective of this longitudinal observational study was to investigate the effects of environmental conditions on calf growth using naturally occurring environmental conditions with the same management regime throughout. This is in contrast to studies such as that by Cockram and Rowan [13], conducted under controlled environmental conditions, or where the management of the calves was adjusted. Additionally, as an alternative to using air temperature as the sole environmental parameter, this study aimed to examine the effect of the proportion of hours for which calves were exposed to effective temperatures below their LCT on their DLWG for two management phases: the first days of life when the calf is in an individual hutch (phase 1), and then when the calves are moved into group pens (phase 2). Effective temperature was chosen, as although air temperature is widely used to assess the thermal conditions, other parameters such as relative humidity and wind speed can amplify the perception of high and low temperatures [24].

## 2. Materials and Methods

### 2.1. Calf Housing and Management

The calves used in this study were sourced from the dairy herds at SRUC Dairy Research and Innovation Centre, Crichton Royal Farm, Dumfries, Scotland, and their management followed normal farm management practices. All calves born within the study period were eligible for recruitment into the study, and consisted of dairy (Holstein) and dairy–beef cross (Holstein–British Blue cross, Holstein–Limousin cross, and Holstein–Aberdeen Angus cross) calves. Both male and female calves were eligible for recruitment. In total, 299 calves were eligible for recruitment into the study. A full descriptive summary of the calves can be found in Section 3.1 (Calves—Descriptive).

**Figure 1:**
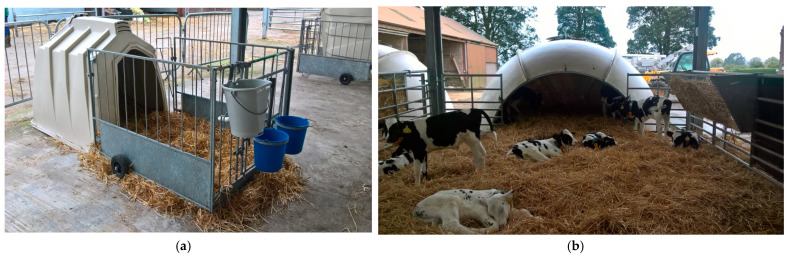
(**a**) Example of the individual calf hutches used in the study; (**b**) example of the group housing igloo pens used in the study.

All calves were removed from their dam within 24 h of birth and given an ear tag for identification, had their navel dipped with an iodine solution, and then were oesophageal tubed with four litres of thawed, quality-tested pasteurised colostrum, and weighed (birthweight). The calves were then taken to the main calf-rearing unit and placed within a straw-bedded individual calf hutch (Calf-Tel Compact, Calf-Tel, Hammel Corporation, Germantown, WI, USA) (Figure 1a), where they had access to fresh water and ad libitum starter pellets (VitaStart + Deccox, ForFarmers, Suffolk, England; crude protein 18%, crude fats and oils 4%, crude fibre 11.5%, crude ash 7%, 3 mm diameter) daily via buckets. From this point on, the calves received three litres of reconstituted milk replacer (Omega Gold, ForFarmers, Suffolk, UK) twice per day (7:30 a.m. and 4:00 p.m., approximately) via individual buckets with teats whilst in their individual hutches. The milk replacer consisted of 23% crude protein, 18% crude fat, 0.1% crude fibre, and 8.5% crude ash, and was fed at a concentration of 15%. All of the buckets for each calf remained with that calf for the duration of time for which it remained in the individual calf hutch. As a matter of routine, all calves received 4 mL Halocur^®^ (Intervet International, Boxmeer, The Netherlands) for their first six days of life due to the history of cryptosporidiosis on the farm. Once calves were able to suckle from the teat confidently, and were assessed to be strong, healthy, and above 6 days of age, the calves were eligible to move into the group housing igloo pen (Figure 1b).

**Figure 2:**
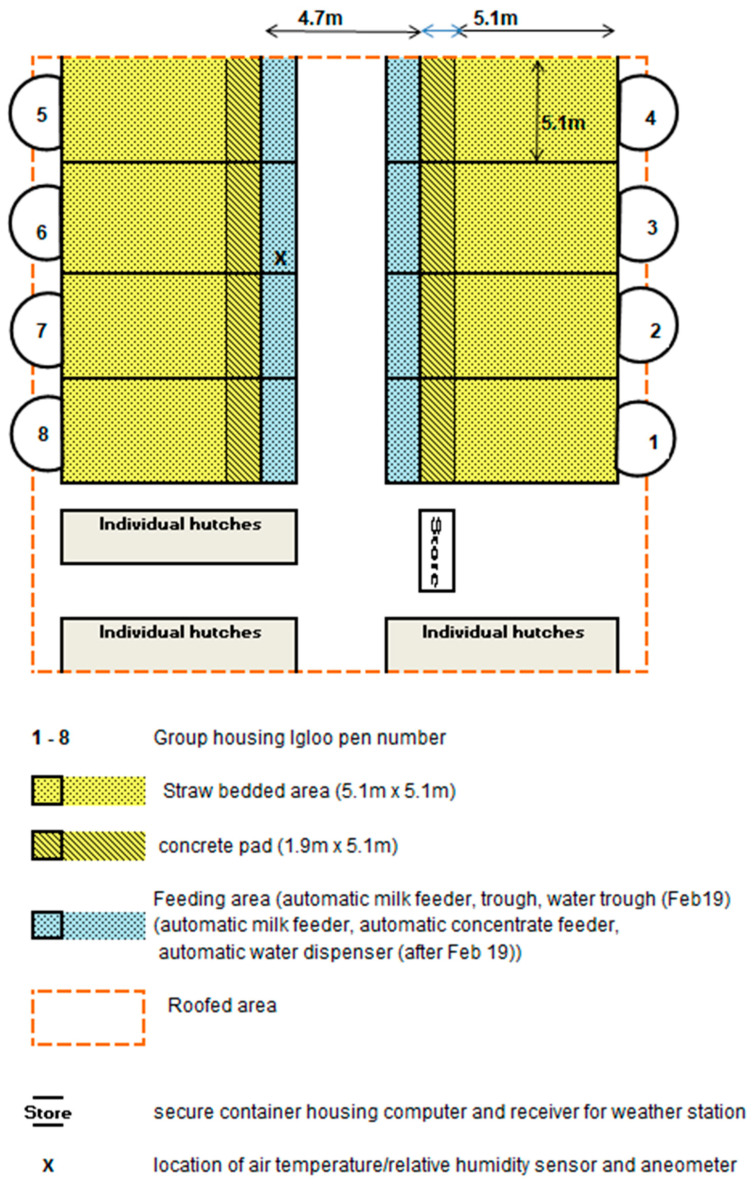
Illustration of the layout of the calf-rearing facility used in the study, featuring locations of individual hutches and group housing igloo pens.

The group housing igloo pen consisted of a hand-laminated fibreglass constructed dome (igloo) (Holm and Laue, Westerfield, Germany) (3.9 m, 4.4 m, 2.2 m: length, width, and height, respectively; volume: 20 m^3^). In front of the igloo there was a roofed pen (5.1 m × 5.1 m, length and width). The flooring of both areas was covered in straw. New straw was added weekly, and all bedding was removed and replaced every 2 weeks. The main calf-rearing unit consisted of 2 rows of 4 group igloo pens (Figure 2). Every pen contained an automatic milk feeder, from which every calf was allowed up to 7.2 L (7 L plus 0.2 L carry-over allowance) of reconstituted milk replacer every day; this was fed at a concentration of 15%.

Upon leaving the individual hutch and entering the group housing igloo system, every calf received 2 mL Rispoval^®^ RS + PI3 IntraNasal (Zoetis, Brussels, Belgium). It was normal practice for the farm to move calves into the group housing igloo pen between 6 and 14 days of age.

### 2.2. Climate Data

Air temperature (°C), relative humidity (%), and wind speed (m/s) were automatically recorded hourly throughout the study period using a Ventus W831 Weather Station (NSH NORDIC A/S, field 4, DK-8740 Brædstrup, Denmark). A sensor to measure air temperature and relative humidity and an anemometer to measure wind speed were located in the central passage of the calf shed at 0.8 m and 1.5 m, respectively. The data from both sensors were downloaded twice per week.

### 2.3. Measurement

For this study, measurements were taken for two management phases: Phase 1 covered the period from when the calves went into the individual hutch after birth until they left it and went into the group housing igloo pen; this phase is hereafter referred to as “B2G” (birth-to-group). Phase 2 covered the period from when the calves left the individual hutch and entered the group housing igloo pen until the end of the study; this phase is hereafter referred to as “G2E” (group-to-end).

Data on date of birth, birthweight (kg), calving ease, and parity of dam were collected from farm records. The calf’s date of birth was used to define its season of birth (winter (December, January, February), spring (March, April, May), summer (June, July, August), autumn (September, October, November)). Data on air temperature (°C), wind speed (m/s), and relative humidity (%) for the period from when the calf went into the individual hutch until it left the individual hutch were also collected retrospectively from the weather station in the calf shed.

#### 2.3.1. Individual Hutch (B2G)

Calves were weighed when they were removed from their dam using a manually operated calibrated weigh crate with Tru-Test digital load cells and an EziWeigh5 weigh head attached (Ritchie Agriculture, Forfar, Angus, Scotland); this weight was referred to as “birth weight”. It was not noted whether or not the calf was weighed after or prior to colostrum consumption, as this information was unable to be retrieved. The calves were weighed again, using the same weigh crate, at the point of leaving the individual hutch and entering the group housing igloo pen (LH weight). Daily liveweight gain (DLWG) (kg/d) for B2G was calculated by dividing the weight gain by the number of days between birth weight and group housing igloo pen entry weight. Calf-level health treatments were collected from farm records, and calves were classified as either having received treatment or not during this period (No, Yes).

#### 2.3.2. Group Housing Igloo Pen (G2E)

Whilst in the group housing igloo pen, there were two recording days per week—typically Mondays and Thursdays (12:30 p.m.–2:00 p.m.), and occasionally on other days where circumstances intervened. Various measurements were taken from each of the calves on these days, as described below.

##### Liveweight

A record of liveweight (kg) was taken for each calf on the recording days. This weight was assigned a weighing number to represent whether this was the calf’s first, second, third, etc. recording of liveweight whilst in the group housing igloo pen for the duration of the study (WGT1, WGT2, WGT3, WGT4, WGT5, WGT6, WGT7). Daily liveweight gain (DLWG) (kg/d) for G2E was calculated but, to take into account the possible changing rate of DLWG over time between entering the group housing igloo pen and the end of study period, a linear regression was applied for G2E, with the value of the slope used as DLWG [9,25].

##### Health Assessment

A health assessment of each calf was made on the day the calf left the individual hutch, and on each recording day, using the Wisconsin method [26,27]. This was carried out by the same trained operator (D.J.B.). This method involved taking a rectal temperature, visually assessing ocular and nasal discharge, head/ear positioning, and the presence or absence of a cough. Each aspect was given a score on a scale of 0–3, with 0 being described as “normal” and 3 as “severe”. A sum of these scores represented the overall health score for the calf, with the lowest possible score being 0 and the maximum score being 15. For this study, rectal temperature was taken using a digital thermometer (Genia Digiflash, Saint-Hilaire-de-Chaléons, France). A score for faecal consistency was not able to be carried out, as the calves were group housed and, thus, faeces from individuals could not be identified, so this was not included in the analyses of health status.

From the scoring, calves were defined as either “healthy”, “diseased”, or “intermediate” based on the criteria in Table 1.

Health status for the calf was then categorised for the G2E period of the study into “ever showed clinical or mild signs of disease” (Intermediate and Diseased—Signs of disease, Yes) or “never showed any signs of disease” (Healthy—Signs of disease, No).

Farm staff involved in the care of the calves were made aware of the results of the health assessments, and treatment was administered at the discretion of the farm. Calf-level treatments were collected from farm records, and calves were classified as either having received treatment or not during this period of the study (No, Yes).

##### Milk Intake Data

Milk consumption data (quantity of calf milk replacer (CMR) consumed each day) were collected for each calf for the period during which it was in the study (from entering the group igloo pen to the last time it was weighed). The milk-feeding equipment was changed within the study period. Initially, calves were fed from an H and L100 (Holm and Laue, Westerfield, Germany—183 calves), and latterly from a BioControl milk feeder (BioControl AS, Grimstad Gård, N-1890 Rakkestad, Norway—116 calves). No calf was fed using both milk-feeding systems.

The number of days from the calf entering the group pen until the last day of measurements being taken was calculated and used to determine the average daily intake of milk (l/d) and CMR (g/d) for each calf. Only average daily CMR intake was used in the analysis.

#### 2.4. Data Analysis

##### 2.4.1. Inclusion and Exclusion Criteria

For calves to be included in the analysis of the B2G stage, they must have had a birthweight recorded and been 14 days of age or less when they left the individual hutch and entered the group housing igloo pen. As well as these criteria, for calves to be included in the analysis of the G2E stage, they also must not have been sold or died before reaching the end of the study period, must have remained in the group housing igloo pen for the full duration of the study period, and must have had a full set of data for milk intake from the automatic milk feeder.

##### 2.4.2. Proportion of Hours Below LCT

Based on the hourly measurements taken from the weather station, the effective temperature (°C) for every hour was calculated using the etv function from the “ThermIndex” package in R [28]. This function incorporates air temperature, relative humidity, and wind speed, and is based on the equation by Suping et al. [29]. The lower critical temperature (LCT) was calculated per day per calf from birth (day 0) to the day they left the study. LCT at birth (day 0) was defined as 15 °C [11], and decreased with the age of the calf [18] by 0.5 °C per day.

The proportion of total hours for which each calf experienced an effective temperature below this LCT was then calculated; this was done by calculating the number of hours for which the effective temperature was below that of the associated age-related LCT for each calf, and then dividing that by the total number of hours for which the calf was in each phase of the study (individual hutch (B2G) and group igloo pen (G2E)). The proportion of hours below the LCT was then categorised into quartiles based on the examination of the distribution plots: B2G (≤0.32, 0.33–0.58, 0.59–0.96, ≥0.97), G2E (≤0.01, 0.02–0.06, 0.07–0.27, ≥0.28).

##### 2.4.3. Statistical Analysis

All statistical analyses were conducted using R software [29]. Datasets were compiled for the two time periods of interest for the study: birth to group pen entry (B2G), and group pen entry until the end of the study period (G2E). Each individual calf was regarded as the experimental unit within both datasets.

Table 2 shows which variables were considered in the analysis of each dataset.

Any calves missing a birth weight were excluded, as their DLWG could not be calculated. As it was normal practice for the farm to move calves into the group housing igloo pen between 6 and 14 days, calves older than 14 days were excluded. Moreover, calves older than 14 days were likely to have experienced a health-related issue in early life. Other reasons for exclusion included the sale or death of the calf before the end of the study period, the return of the calf to an individual hutch after initially entering the group housing igloo pen, milk consumption data being unavailable, or only having one weight in the group housing igloo pen. The dependent variable—DLWG—was investigated for association with the independent variables mentioned in Table 2.

To analyse the dataset for the time period of B2G, multiple univariable linear models using the lm function in R [30] were used to screen the independent variables for level of significance.

To analyse the G2E dataset, multiple univariable linear mixed-effects models with random effects for the group in which the calf was placed, the group igloo pen in which they were placed, and the milk-feeding system used were constructed. The lmer function from the “lme4” package [31] was used. For both sets of analyses, independent variables that had a *p*-value of less than 0.20 were carried forward and included in a multivariable model.

A maximal model was constructed and then optimised using backward step selection until only variables significant at *p* < 0.05 remained. Tukey’s post hoc test was used to obtain significance between factor levels of significant variables only.

## 3. Results

### 3.1. Calves—Descriptive

In total, 299 calves were enrolled onto the study. Of these, 226 were dairy calves (137 female, 89 male) and 73 were dairy–beef cross calves (34 female, 39 male). Of these 299 calves, 109 were born from primiparous dams and 190 from multiparous dams. In terms of calving ease, 263 had an unassisted birth and 36 had an assisted birth. Out of the 299 calves, 80 were born in winter (26 dairy, 54 dairy–beef cross), 85 in spring (76 dairy, 9 dairy–beef cross), 54 in summer (45 dairy, 9 dairy–beef cross), and 80 in autumn (51 dairy, 29 dairy–beef cross). One calf was excluded from the data due to a missing birth weight, and 27 were excluded as they were older than 14 days when moved into the group housing igloo pen. Data from the remaining 271 calves were used in the analysis of the B2G dataset.

In terms of the age at which these 271 calves left the individual hutch and entered the group housing igloo pen, 19 calves were 6 d, 52 calves were 7 d, 45 calves were 8 d, 45 calves were 9 d, 29 calves were 10 d, 28 calves were 11 d, 27 calves were 12 d, 16 calves were 13 d, and 10 calves were 14 d.

Of the 271 calves during the B2G stage, 37 received treatment whilst in the individual hutches (19 diarrhoea (6.1 d ± 2.05), 13 respiratory disease (6.1 d ± 2.36), and 5 other disease (3.6 d ± 1.63) (mean age at treatment ± SD). During the G2E stage, of the 221 calves, 95 calves were treated for 110 incidents (36 scour (14.5 d ± 4.58), 62 respiratory disease (22.0 d ± 6.46), and 12 for other health related reasons (18.8 d ± 5.97) (mean age at treatment ± SD). Of the 95 calves treated during this period (G2E), 12 were treated for more than one disease. A total of 16 calves were treated in both the B2G and G2E stages.

Five calves died or were euthanized during the study (one experienced a seizure, two suffered traumatic leg injuries, one had an umbilical abscess, and one had an injury to an eye that was refractory to treatment) and one calf was sold before completing the study. Three calves were also excluded as they returned to the individual hutches from their group igloo pen. There were two separate occasions throughout the study where milk-feeding data could not be recovered as a result of power failure; this affected 41 calves, which were excluded from the study. Following exclusions, the dataset used for the analysis of the G2E time period contained 221 calves.

The calves, on average, lost weight (mean DLWG −0.07kg/d) during the B2G time period, although there was a large variation in DLWG (Table 3). This variation was slightly less for G2E, where the calves had a mean DLWG of 0.60 kg/d. For the G2E period, 98 calves showed no signs of disease (all scores in Healthy category) and 123 showed signs of disease (one or more scores in the Intermediate or Disease categories).

### 3.2. Climate—Descriptive

Over the course of the study, there was a range of climatic conditions experienced by the calves (Table 4). For the time period from birth until leaving the individual hutch (B2G), some calves experienced an effective temperature that was always below their age-related LCT. On average, calves in the individual hutches spent 60% of the time below their LCT.

### 3.3. Daily Liveweight Gain—Birth to Group Pen (B2G)

The final multivariable linear model for the daily liveweight gain of calves from birth until they left the individual hutch and entered the group housing igloo pen (B2G) contained the following variables: the proportion of hours for which the calf was exposed to effective temperatures below its age-related LCT (ProphrsLCT), the weight of the calf at birth (Birth weight), and the age at which the calf left the individual hutch (Age leaving individual hutch) (Table 5). No other variables were significant in the model (Season of birth: *p* = 0.454; Parity of dam at birth: *p* = 0.755; Sex of calf: *p* = 0.929; Breed classification: *p* = 0.391; Calving ease: *p* = 0.142; (Farm) Treatment administered: Individual hutch: *p* = 0.273). A potential reason for these variables being non-significant could be the management of the calves throughout the study.

**Figure 3:**
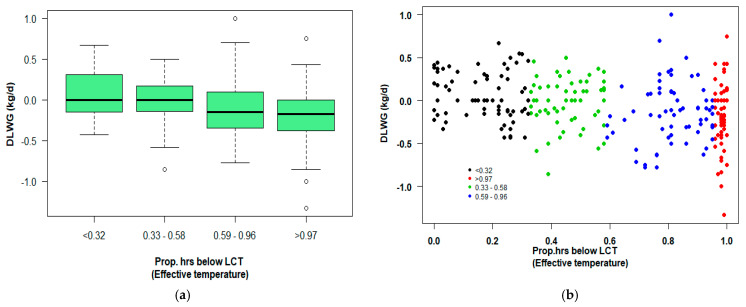
(**a**) Effect of proportion of hours for which effective temperature was below LCT on daily liveweight gain (DLWG, kg/d) for the period between birth and entering the group housing igloo (B2G); (**b**) scatterplot of daily liveweight gain (DLWG, kg/d) and the proportion of hours for which effective temperature was below LCT.

There was a significant effect of ProphrsLCT on DLWG (B2G) (F (3, 265) = 6.098, *p* < 0.001). There was a significant difference found between the categories ≤ 0.32 and 0.59–0.96 (*p* = 0.015), ≤0.32 and ≥0.97 (*p* < 0.001), and 0.33–0.58 and ≥0.97 (*p* = 0.041). The results suggest that the greater the proportion of time that a calf spent below the LCT, the greater the reduction in DLWG (Figure 3).

The birth weight of the calf had a significant effect on DLWG for the period between birth and leaving the individual hutch (F (1,265) = 35.154, *p* < 0.001). When accounting for all other variables in the model, for every kg increase in birth weight, DLWG (B2G) reduced by 0.02 kg/d, indicating that heavier calves grew more slowly during this period.

The age at which the calf left the individual hutch also had a significant effect on the daily liveweight gain (DLWG, kg/d) (F (1,265) = 11.196, *p* < 0.001); DLWG (B2G) increased by 0.03kg/d for every day older the calf was when leaving the individual hutch.

### 3.4. Daily Liveweight Gain—Entering Group Pen until End of Study Period (G2E)

The final model examining factors affecting the daily liveweight gain of calves for the G2E period of the study contained the proportion of hours for which the calf was exposed to effective temperatures below its age-related LCT (ProphrsLCT), the age of the calf on entry to the group pen (Entry age), and its average daily intake of CMR (CMR intake) (Table 6).

There was no significant effect of ProphrsLCT on daily liveweight gain (G2E) (chisq = 2.747, 3 df, *p* = 0.432).

The age at which calves entered the group housing igloo pen also had a significant effect on DLWG for G2E (chisq = 6.343, 1 df, *p* = 0.012); the final model indicated that for every day older the calf was on entry to the group pens, their DLWG (G2E) increased by 0.01 kg/d.

**Figure 4:**
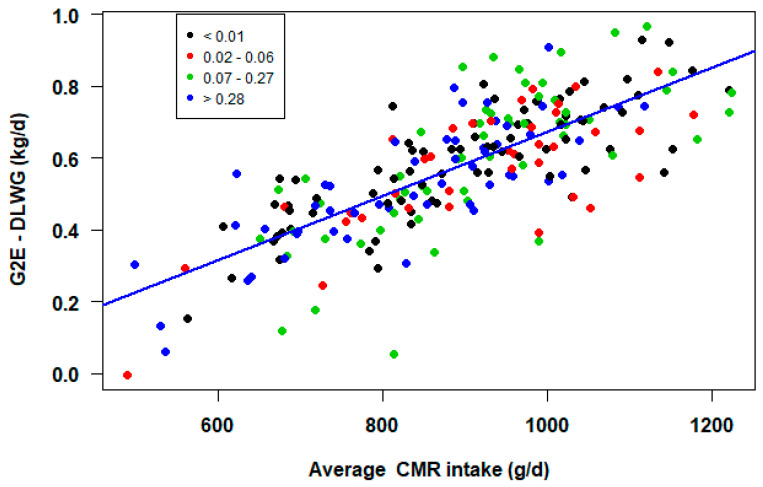
Effect of average CMR intake (g/d) on daily liveweight gain (DLWG, kg/d) between entering the group housing igloo pen and the end of the study period (G2E) by proportion of hours below LCT (<0.01, 0.02–0.06, 0.07–0.27, >0.28).

There was a significant effect of the average CMR intake on the daily liveweight gain for G2E (chisq = 348.686, 1df, *p* < 0.001). Furthermore, there was a significant positive correlation between average CMR intake and DLWG (G2E) (r = 0.77, *p* < 0.001) (Figure 4); the more CMR consumed by the calf, the higher the growth rate achieved.

## 4. Discussion

The DLWG values measured for the calves in this study were similar to those reported in other UK studies. Bazeley et al. [32] reported that there was no weight gain in calves in their first eight days of life, which was similar to this study, which showed a mean DLWG for B2G of −0.07 kg/d, although the range was −1.33 to +1.00 kg/d. The range of DLWG shows that it is possible for calves to have a positive DLWG in their early stages of life. For the G2E time period, the mean DLWG was 0.60 kg/d for calves 29 days of age (mean age at end of study). The DLWG values for the present study were slightly higher than those reported for the top performing herds in the study of Bazeley et al. [32] (0.52 kg/d) and the non-jacketed calves in the study of Scoley et al. [33]. Differences in the housing and management systems on each of the farms in these studies will likely account for these minor differences in reported growth rates.

### 4.1. Proportion of Hours Below LCT

In the present study, there was a significant effect of the proportion of hours for which the calves were exposed to effective temperatures below their LCT on their daily liveweight gain (DLWG, kg/d) when in the individual hutch from birth until leaving the individual hutch and entering the group housing igloo pen. However, this had no significant effect on DLWG for the older age group in the time period from entering the group housing igloo pen until the end of the study (~28 days of age). An important point worth noting is how rapidly the vulnerability of calves changes with age, which is often a message that the industry often fails to appreciate. For this study, the proportion of hours for which the calves were exposed to effective temperatures below their LCT during the B2G period was split into quartiles (≤0.32, 0.33–0.58, 0.59–0.96, >0.96), as it was for the G2E period (<0.01, 0.02–0.06, 0.07–0.27, >0.28). The B2G period covered the period from birth until leaving the individual hutch (between 6 and 14 days of age) and the period G2E covered the period from entering the group housing igloo pen (between 6 and 14 days of age) until the end of the study (approximately 28 days of age). It can be seen from the quartiles from both time periods that the older calves (G2E period) were not exposed to effective temperatures below their LCT to anywhere near the same extent as they were during the B2G period.

### 4.2. Climatic Environment Parameters

The proportion of hours below age-related LCT and/or effective temperatures are not the conventional climatic environmental parameters used to assess thermal conditions. In some studies, the temperature–humidity index (THI) has been used. Shivley et al. [34] found that calves exposed to a THI of less than 50 during the pre-weaning period had a higher DLWG compared to calves exposed to a THI between 50 and 59 or greater than 70. However, it is suggested that the THI is more of an indicator of heat stress rather than of general conditions in cooler climates. Moreover, the THI is based solely on a combination of air temperature and relative humidity; it does not take wind speed into consideration, which effective temperature does. The housing for the calves in the present study was within an open-sided umbrella-like structure and, therefore, the calves had the potential to be exposed to wind; thus, the use of the effective temperature was thought to be most appropriate. As Hahn et al. [35] state, there are limitations to the use of air temperature as a representation of the thermal environment, and a combination of parameters should be used. There is a limitation in the work presented as well, as the calculation of effective temperature did not take the radiant heat from sunlight into consideration and, therefore, was not completely representative of the heat exchange with the environment. Some work has been carried out to examine the appropriateness of human comfort indexes for use in livestock—particularly heat stress [36]—but further work should be carried out to develop a more general index for livestock, in particular for calves.

### 4.3. B2G Period

For the B2G period, it is likely that the young calves struggled to acclimatise to the environment in which they were kept. According to Nienaber and Hahn [37], this process can take days or even weeks to be achieved. Rowan [38] reported that acclimatisation develops with the age of the animal. Another possible explanation for this result could lie within the calculation used for the proportion of hours below the LCT. It is acknowledged that the calculation used for the proportion of hours below the LCT is imperfect, but this calculation is a significant improvement on previous studies that merely measured air temperature, as it at least takes into account the influence of the age of the calf on its LCT. There would be very few calves who were exposed to effective temperatures that were above their age-related LCT on an hourly basis for the individual hutch period. Most calves in Scotland, including those in the present study, would be exposed to effective temperatures at some stage during the day—but mostly at night—that were below this LCT in winter months. As this study has demonstrated, calves that were exposed to a high proportion of hours below their LCT had significantly lower DLWG. The calves were offered a high-volume, energy-dense milk diet during this management stage (B2G) (6 L/d at 15% concentration which equated to 900 g CMR/d), regardless of season of birth. Therefore, despite this, the climatic environment still had an impact on the performance of these calves. However, milk intake was not recorded during this management stage, and it was assumed that calves drank all of the milk that was offered to them at both feedings. Therefore, this result suggests that the period following birth is when the calf is most vulnerable to the climatic environment, and management procedures such as the application of calf jackets could potentially be beneficial by acting as a barrier to reduce heat loss to the environment [39].

### 4.4. G2E Period

The non-significant effect of proportion of hours below LCT on DLWG for the time period from entering the group housing igloo pen until the end of the study period (G2E) could be as a consequence of the calves’ development. By this stage, their LCT was below the average climatic conditions for the region. The calves in the present study entered the group housing igloo pen when their age-related LCT was 10 °C (mean entry age was 10 days). Every day after this, their LCT declined by 0.5 °C and, therefore, there would be a higher chance that the calf would not be below its LCT unless there had been a sudden dramatic change in environmental conditions—especially in southern Scotland, where the mean daily air temperature has been estimated to be around 9 °C [40]. Another reason is related to the behavioural response of the calves. For the G2E time period, the calves were group housed, whereas for B2G they were housed individually. One behavioural response to low environmental temperatures is huddling [17]. Once in the group housing igloo pen, calves had the opportunity to keep warm by huddling with the other calves in the group, whereas in the individual hutch, each calf was reliant on other processes, such as the ability to nest in the bedding material to maintain warmth. Although not recorded in the present study, it would have been of interest to see whether this behavioural response (huddling) was occurring, and in particular whether it occurred during specific times of the day (e.g., around dawn and overnight) and its relationship with the climatic environment.

### 4.5. Birth Weight

It was found that birth weight had a significant effect on DLWG for the period from birth until leaving the individual hutch (B2G). Donovan et al. [41] and Yaylak et al. [42] also both found that birth weight had a significant influence on subsequent daily liveweight gain. However, daily liveweight gain for both of these studies was recorded over longer periods of time than in the present study (birth to 6 months and birth to weaning, respectively). This effect of birth weight on subsequent growth rate may be a result of the volume of milk given to the calves during this early phase of life. When they were housed in the individual hutch (B2G), calves in the present study were given 6 L/d of milk. In the case of the lightest calf birth weight (31 kg), this equated to nearly 20% of bodyweight, whereas for the heaviest calf (67 kg), the 6 L/d only equated to 8.9% of bodyweight. Therefore lighter born calves potentially had more available energy, over and above that was required for maintenance, which could be used for growth. This explanation is supported by the results of Khan [43], who showed that calves from 3 d of age can safely consume 20% of their bodyweight per day, and that an increase in consumption supports an increase in DLWG.

### 4.6. Age Leaving Individual Hutch and Entering Group Houisng Igloo Pen

The age at which the calf left the individual hutch and entered the group housing igloo pen had a significant effect on DLWG in both the B2G and G2E periods. For the B2G time period, the older the calf was upon leaving the individual hutch and entering the group pen, the higher the DLWG. The same trend was evident for the G2E time period, with the greater the age at entry to the group pen, the higher the DLWG. A factor that could have influenced this result is the use of the automated feeders, and the possibility that older calves take less time to learn to use them. Fujiwara et al. [44] reported that calves that took a considerable time to learn and adapt to the automated feeders had reduced milk intake and poor growth rate in the initial weeks of entering the group pen. They also reported that calves introduced around 6 days of age took longer to voluntarily consume milk from the automated feeders than calves at 9 days of age. This result was also found in the study by Jensen [45].

### 4.7. CMR Intake

The amount of CMR consumed per day had a significant effect on DLWG for the G2E time period. This result is consistent with those found in other studies [2,22,23,46]. Morrison et al. [21] showed that an increasing level of milk replacer produced a higher DLWG between 0 and 28 days of age, while Johnson et al. [46] found that there was a positive correlation of CMR intake with daily liveweight gain. The study by Khan et al. [43] demonstrated that calves can safely be fed 20% of their bodyweight per day, which can result in an increase in DLWG. These results could be due to the extra energy that the increase in CMR intake provides above maintenance energy. Further work could consider examining the proportion of maintenance energy provided by the daily CMR intake, in terms of being above and below that which is required by the calf, to validate this theory.

### 4.8. Strategies for Achieving Thermal Comfort

There are a few farm-level practices that could assist with achieving thermal comfort other than feeding more energy [18]; one such practice would be the application of calf jackets. Scoley et al. [33] reported a 6.37 °C increase in the skin temperature of calves that received calf jackets compared to calves that did not receive a calf jacket when the ambient air temperature was 7.7 °C (mean of their study). Rawson et al. [47] concluded that there was a 52% increase in whole-animal insulation when calves wore a calf jacket at extremely low ambient temperatures. However, as Robertson [39] conveys, there is still a gap between the scientific evidence and the popularity as well as the benefit of use of calf jackets.

Another practice could be to apply more bedding material to the housing in order to enable the calf to “nest”. Nesting behaviour is when the calf tries to bury itself within the bedding material, hence creating a nest-like feature. Nesting scoring can be carried out as a quantification of thermal comfort, with an example of its use being captured by Lago et al. [48].

## 5. Conclusions

The present study has shown that the key performance indicator of daily liveweight gain is affected by the climatic housing environment in the very early stages of the rearing phase of the calf. Therefore, emphasis should be placed upon the management of the calf at this stage, and providing an appropriate plane of nutrition will assist with achieving target daily liveweight gain. The application of amelioration strategies may also be beneficial, especially in times when temperatures are below the LCT. Further investigation should be carried out to examine the various comfort indices and assess which is the most suitable for use with pre-weaned calves and different types of housing. It would be hoped that other studies would consider using hours below LCT and reject the use of average environmental values as a variable for analysis.

## Figures and Tables

**Table 1:** Calf health status definitions based on the Wisconsin health scoring method.

Health Status	Signs of Disease	Definition Criteria
Healthy	No	Rectal temperature score ≤ 2 with an overall Wisconsin score ≤ 3
Intermediate	Yes	Rectal temperature score ≤ 2 with an overall Wisconsin score = 4
Diseased	Yes	Rectal temperature score = 3 regardless of overall Wisconsin score; Rectal temperature score ≤ 3 with an overall Wisconsin score ≥ 5

**Table 2:** Inclusion of variables of interest in the two datasets (birth to leaving the individual hutch (B2G); entering the group housing igloo pen until end of the study period (G2E)).

Variable	B2G	G2E
Sex of calf (male, female)	✓	✓
Breed classification (dairy, dairy–beef cross)	✓	✓
Calving ease (assisted birth, unassisted birth)	✓	✓
Parity of dam at birth (primiparous, multiparous)	✓	✓
Season of birth (winter, spring, summer, autumn)	✓	✓
Birth weight (kg)	✓	✓
(Farm) Treatment administered—Individual hutch (no, yes)	✓	✓
Age at leaving individual hutch/entering group housing igloo pen (d)	✓	✓
(Farm) Treatment administered—Group housing igloo pen (no, yes)		✓
Signs of disease (based on Wisconsin score) (no, yes)		✓
Mean quantity of CMR consumed (g/d)		✓
Proportion of hours below LCT (effective temperature)	✓	✓
Daily liveweight gain (kg/d)	✓	✓

CMR—calf milk replacer., LCT—lower critical temperature.

**Table 3:** Description of calf-related parameters by phase: individually housed in hutches (B2G), and whilst housed in group housing igloo pens (G2E).

Parameter	Mean	SD	Median	Min	Max
**B2G**					
Birth weight (kg)	43.2	6.2	42.0	31.0	67.0
LH weight (kg)	42.7	5.8	42.0	29.0	65.0
Age leaving individual hutch (d)	9.3	2.2	9.0	6.0	14.0
DLWG (kg/d)	−0.07	0.34	−0.08	−1.33	1.00
**G2E**					
Birth weight (kg)	43.3	6.2	42.0	31.0	67.0
Group pen entry age (d)	9.1	2.2	9.0	6.0	14.0
Entry weight (kg)	42.9	5.9	42.0	29.0	65.0
End weight (kg)	55.1	7.3	54.0	38.0	78.0
End age (d)	29.5	1.2	30.0	25.0	32.0
DLWG (kg/d)	0.60	0.20	0.60	0.00	1.00
Average CMR intake (g/d)	890.5	152.3	909.6	489.8	1223.7

LH weight—weight upon leaving individual hutch; DLWG—daily liveweight gain; CMR—calf milk replacer.

**Table 4:** Description of climate parameters to which calves were exposed over the duration of the study.

Parameter	Mean	SD	Median	Min	Max
Air temperature (°C) ^1^	10.3	5.2	10.0	−3.9	26.7
Wind speed (m/s) ^1^	0.2	0.4	0.0	0.0	3.0
Relative humidity (%) ^1^	81.1	11.3	84.0	27.0	99.0
Effective temperature (°C) ^1^	11.3	5.1	11.3	−5.3	24.2
Proportion of hours effective temperature < LCT (B2G) ^2^	0.60	0.33	0.58	0.00	1.00
Proportion of hours effective temperature < LCT (G2E) ^3^	0.15	0.17	0.06	0.00	0.61

LCT—lower critical temperature; B2G—period from birth until leaving individual hutch; G2E—period from entering group housing pen until the end of the study period. ^1^ Based on data from the day the 1st calf recruited to the study was born until the date the last calf was weighed on study. ^2^ Based on data for 271 calves. ^3^ Based on data for 221 calves.

**Table 5:** Final model describing variables affecting daily liveweight gain (DLWG) from birth until entering the group housing igloo pen (B2G).

Daily Liveweight Gain (kg/d)—B2G					
Variable	Level	Estimate	SE of Estimate	*p*-Value for Reference	*p*-Value for Effect
Intercept		0.537	0.153		<0.001
ProphrsLCT	≤0.32	Reference			<0.001
	0.33–0.58	−0.061	0.052	0.240	
	0.59–0.96	−0.157	0.052	0.003	
	≥0.97	−0.199	0.052	<0.001	
Birth weight (kg)		−0.018	0.003		<0.001
Age leaving hutch (d)		0.028	0.009		<0.001

ProphrsLCT—proportion of hours for which effective temperature was below lower critical temperature.

**Table 6:** Final mixed model describing variables affecting daily liveweight gain (DLWG) from entering the group housing igloo pen until the end of the study period (G2E).

Daily Liveweight Gain (kg/d)—G2E					
Variable	Level	Estimate	SE of Estimate	*p*-Value for Reference	*p*-Value for Effect
Intercept					<0.001
ProphrsLCT	≤0.01	Reference			0.432
	0.02–0.06	−0.037	0.025	0.132	
	0.07–0.27	−0.008	0.027	0.776	
	≥0.28	0.002	0.031	0.958	
Entry age (d)		0.009	0.004		0.012
Average CMR intake (g/d)		0.001	0.000		<0.001

ProphrsLCT—proportion of hours for which effective temperature was below lower critical temperature; CMR—calf milk replacer. Daily liveweight gain (DLWG) for the G2E period (period from entering the group housing pen until the end of the study period) was calculated through the application of a linear regression.

## Data Availability

The data presented in this study is available upon request from the corresponding author.
